# Draft genome sequences of four Newcastle disease virus isolates from commercial poultry farms in Ethiopia

**DOI:** 10.1128/mra.00359-25

**Published:** 2025-08-27

**Authors:** Getnet Molla, Demsachew Guadie, Dawit Hailu Alemayehu, Abde Aliy Mohammed, Abaysew Ayele, Keyru Tuki, Molalegne Bitew

**Affiliations:** 1 Institute of Biotechnology, Addis Ababa University 37602, Addis Ababa, Ethiopia; 2 College of Veterinary Medicine, Jigjiga University 360634, Jijiga, Ethiopia; 3 Armauer Hansen Research Institute (AHRI) 70605, Addis Ababa, Ethiopia; 4 Animal Health institute (AHI), Sebeta, Ethiopia; 5 Health Biotechnology Directorate, Bio and Emerging Technology Institute 504847 , Addis Ababa, Ethiopia; Katholieke Universiteit Leuven, Leuven, Belgium

**Keywords:** comple genome, NDV, Ethiopia, sequencing

## Abstract

Genome sequencing of four Newcastle disease virus (NDV) isolates from vaccinated chickens in Central Ethiopia, North Wollo, and Shewa (April 2023–June 2024) revealed highly virulent strains, belonging to newly identified subgroups within genotype VII.

## ANNOUNCEMENT

Newcastle disease (ND) is a highly contagious viral disease in poultry, caused by the Newcastle disease virus (NDV), a member of the *Avulavirus* genus in the *Paramyxoviridae* family. NDV has a single-stranded RNA genome encoding six major structural proteins: nucleocapsid (NP), phosphoprotein (P), matrix (M), fusion (F), hemagglutinin-neuraminidase (HN), and large polymerase (L), along with two non-structural proteins (1). Genome diversity is observed with lengths of 15,186, 15,192, or 15,198 nucleotides, classified into Class I and Class II genotypes (2–4). Class II strains pose significant threats to poultry, particularly in Ethiopia, where poor vaccine handling exacerbates challenges (5, 6).

In this study, 314 tissue and swab samples were collected from vaccinated commercial chickens displaying suspected clinical signs of ND during an outbreak from April 2023 to June 2024 in Central Ethiopia, North Wollo, and Shewa. The samples were pooled into 63 composite samples and processed at the Animal Health Institute, where viral RNA was extracted using the QIAamp Viral RNA Mini Kit. NDV infection was confirmed in 11 pooled samples via qRT-PCR (7). Positive samples were inoculated into 11-day-old embryonated eggs for isolation (8). Whole-genome sequencing was performed on five qRT-PCR positive samples with the lowest Ct values using the sequence-independent single primer amplification (SISPA) technique. The SISPA method included reverse transcription of viral RNA into complementary DNA (cDNA), followed by amplification through PCR (9). PCR products were purified, quantified by Qubit 4 fluorometer, and libraries were prepared using ONT Ligation Sequencing Kit (Native Barcoding Kit 96 V14 [SQK-NBD114.96]). Sequencing was conducted for 72 h using MinION Mk1B. The quality of raw reads was assessed (Table 1) using FASTQC (v0.12.1), and adapter contamination was removed using trimmomatic (v0.39). High-quality reads (Phred quality score >20, minimum length 50 bp) were retained. Kmer-based taxonomy with Kraken2 confirmed the reads as NDV, and genome assembly was performed using BWA-MEM against NDV reference genomes (NC_005036.1 and NC_075404.1). Analysis revealed two NDV genomes of 15,192 and two of 15,186 nucleotides, indicating genetic diversity among Ethiopian NDV strains. Annotation was done using Geneious 11.1.5 and ORF finder algorithm.

**TABLE 1:** A general sequence data information

Sample ID	No of raw reads	No of reads after filtering	Coverage bases	Coverage (%)	Mean depth	Mean MapQ	Accession number
NV10.1	69,213	455	14,796	97.4	26.6	57.5	PV189285
NV10.2	80,795	298	14,654	96.5	18.9	41.6	PV189286
NV30787.1	70,208	2402	15,136	99.6	157.6	57.7	PV189287
NV30787.2	101,609	1649	14,970	98.6	122.0	44.7	PV189288

Sequence alignment with representative NDV strains showed the cleavage site motif ^112^RRQKRF^117^, unlike local avirulent vaccine strains (^112^GRQGR↓L^117^), confirming their velogenic nature. The strains were genetically similar to genotype VII (10, 11), with similarities of 97.2%, 94.1%, 97.6%, and 96.3% to an Iranian NDV strain ON184061, and 82.5% to 84.2% to the LaSota vaccine strain AF077761. This indicates the circulation of velogenic NDV strains in Ethiopian poultry, potentially reducing vaccine effectiveness due to antigenic differences.

In conclusion, four virulent NDV strains were isolated, identified as genotype VII.1.1, matching the prevalent genotype reported in Iranian isolates and recognized in East Africa and Asia.

## Data Availability

The nearly complete genome sequences of all isolates were deposited in GenBank with the accession numbers PV189285, PV189286, PV189287 and PV189288. The raw sequencing data has been uploaded to NCBI SRA, and the accession numbers are (SRX29133151, SRX29133152, SRX29133153, SRX29133154).
